# To Check Cough

**Published:** 1874-11

**Authors:** 


How to Check Coughs.—Dr. Brown-Sequard, in his late Boston lectures, says that there are many facts which show that morbid phenomena of respiration can always be stopped by the influence of arrest. Coughing, for instance, can be stopped by pressing on the nerves of the lip in the neighborhood of the nose. A pressure there may prevent a cough when it is beginning. Sneezing may be stopped by the same mechanism. Pressing in the neighborhood of the ear, right in front of the ear, may stop coughing. It is also preventive of hiccough, but much less so than in sneezing or coughing. Pressing very hard on the top of the mouth inside is also a means of stopping coughing. And, he adds, that the will has immense power there. There was a French nurse who used to say: "The first patient who coughs here will be deprived of his food to-day." It was exceedingly rare that a patient coughed then.—*Canada Medical Record.*

The sculptor, Brodie, has nearly completed his colossal statue of Sir. J. Y. Simpson. It represents him in a sitting posture, in a professor's gown, and in the act of lecturing. He is also at work on the bust of the same physician, which will be placed in Westminster Abbey.—*The Clinic.*

